# Supplementary material for: Characterization of Silver Carbonate Nanoparticles Biosynthesized Using Marine Actinobacteria and Exploring of Their Antimicrobial and Antibiofilm Activity
Source: Mar Drugs. 2023 Oct 13;21(10):536. doi: 10.3390/md21100536 (PMC10608482; doi:10.3390/md21100536)
Supplement: Supplementary file 1 [file marinedrugs-21-00536-s001.zip › marinedrugs-2648403-supplementary.pdf]

Supplementary Material

Characterization of Silver Carbonate Nanoparticles Biosynthesized using Marine *Actinobacteria* and Exploring of their Antimicrobial and Antibiofilm Activity

Omar Messaoudi [1,2,*], Ibrahim Benamar [1,2], Ahmed Azizi [3], Salim Albukhaty [4,5,*], Yasmina Khane [6], Ghassan M. Sulaiman [7,*], Mounir M. Salem-Bekhit [8,*], Kaouthar Hamdi [1], Sirine Ghoummid [1], Abdelhalim Zoukel [9,10], Ilhem Messahli [1], Yacine Kerchich [11], Farouk Benaceur [1,12], Mohamed M. Salem [13] and Mourad Bendahou [2]

1 Department of Biology, Faculty of Science, University of Amar Telidji, Laghouat 03000, Algeria; ib.benamar@lagh-univ.dz (I.B.); k.hamdi.bio@lagh-univ.dz (K.H.); goumidsirine@gmail.com (S.G.); messahli.ilhem@gmail.com (I.M.); f.benaceur@lagh-univ.dz (F.B.)

2 Laboratory of Applied Microbiology in Food and Environment, Abou Bekr Belkaïd University, Tlemcen 13000, Algeria; bendahou63@yahoo.fr

3 Department of The Common Trunk Sciences and Technology, Faculty of Technology, University of Amar Telidji, Highway Ghardaia, P.O. Box G37 (M'kam), Laghouat 03000, Algeria; a.azizi@lagh-univ.dz

4 Department of Chemistry, College of Science, University of Misan, Maysan 62001, Iraq

5 College of Medicine, University of Warith Al-Anbiyaa, Karbala, 56001, Iraq

6 Faculty of Science and Technology, University of Ghardaia, BP455, Ghardaia 47000, Algeria; yasminekhane@yahoo.fr

7 Division of Biotechnology, Department of Applied Sciences, University of Technology, Baghdad 10066, Iraq

8 Department of Pharmaceutics, College of Pharmacy, King Saud University, P.O. Box 2457, Riyadh 11451, Saudi Arabia

9 Laboratory Physico-Chemistry of Materials, Laghouat University, Laghouat 03000, Algeria; abdelhalim.zoukel@gmail.com

10 Center for Scientific and Technical Research in Physicochemical Analysis (PTAPC-Laghouat-CRAPC), Laghouat 03000, Algeria

11 École Nationale Polytechnique (ENP), Laboratory of Environmental Science and Technology El Harrach 16200, Algeria; yacine.kerchich@g.enp.edu.dz

12 Research Unit of Medicinal Plant (RUMP) Attached to Center of Biotechnology (CRBt, 3000, Constantine), Laghouat 03000, Algeria

13 Huazhong University of Science and Technology, Wuhan, 430030, China; medozcockney@gmail.com

* Correspondence: o.messaoudi@lagh-univ.dz (O.M.); albukhaty.salim@uomisan.edu.iq (S.A.); ghassan.m.sulaiman@uotechnology.edu.iq (G.M.S.); mbekhet@ksu.edu.sa (M.M.S.-B.)

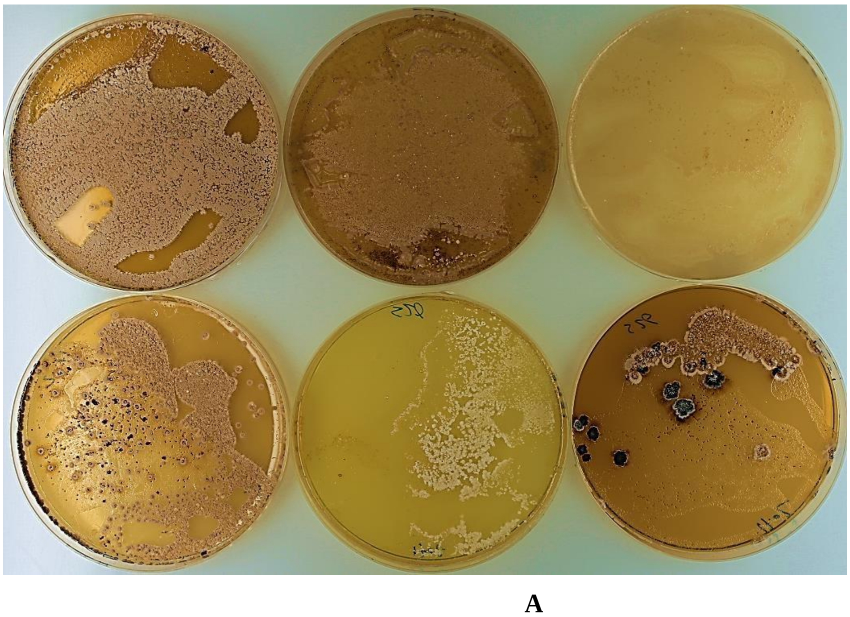

A

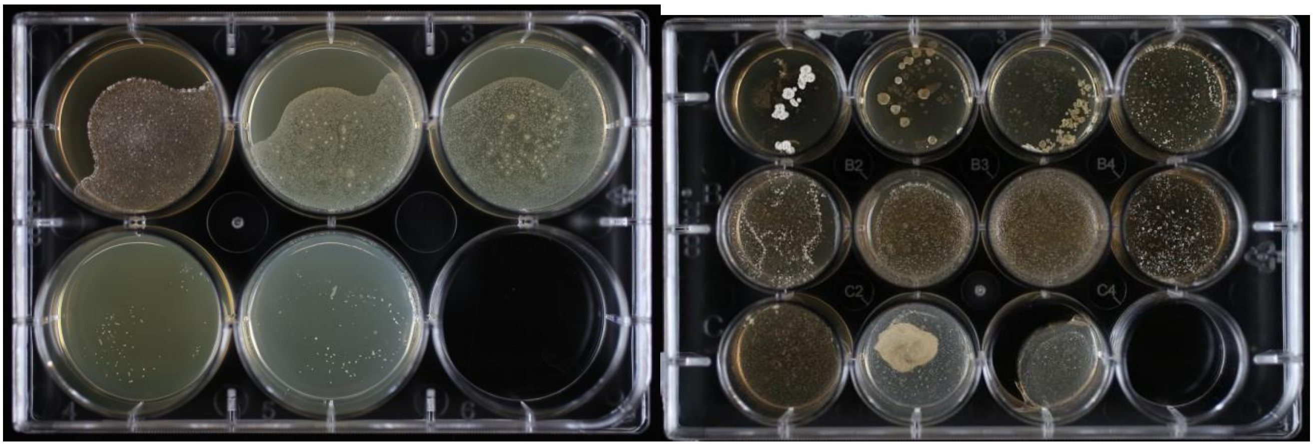

B **C**

Figure S1. A. Colony description and melanin pigment formation; from top left to bottom right: ISP2, ISP3, ISP4, ISP5, ISP6, and ISP7. **B.** Sodium chloride tolerance test; [NaCl] from top left to bottom right : 0%, 2.5%, 5%, 7.5%, 10%. **C.** Carbon utilization test; from top left to bottom right: glucose, arabinose, sucrose, xylose, inositol, mannose, fructose, rhamnose, raffinose, cellulose, water).

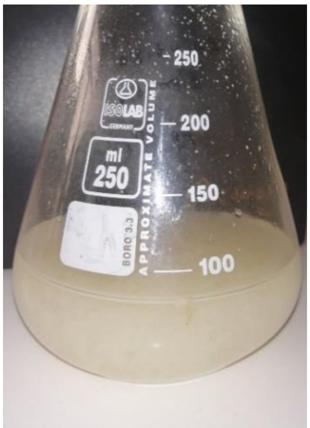

After the addition of $AgNO_3$ to the biomass of strain S26

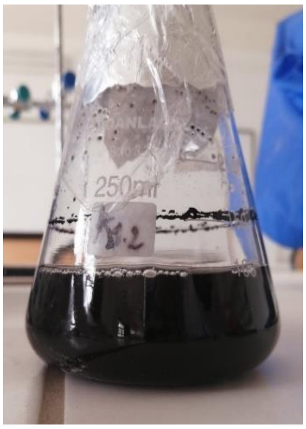

Before the addition of $AgNO_3$ to the biomass of strain S26

Figure S2. Biosynthesis of silver carbonate nanoparticles by the biomass of *Saccharopolyspora erythraea*
S26 strain.

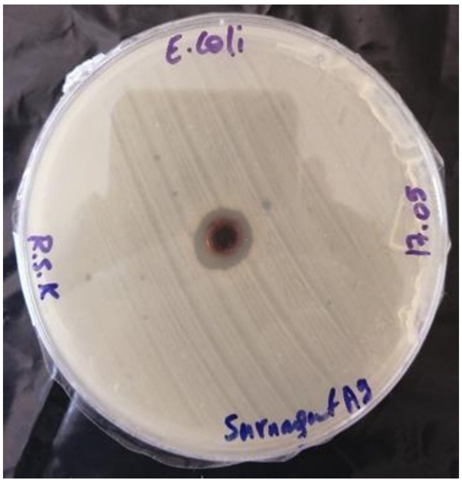

***Escherichia coli* (ATCC 25922)**

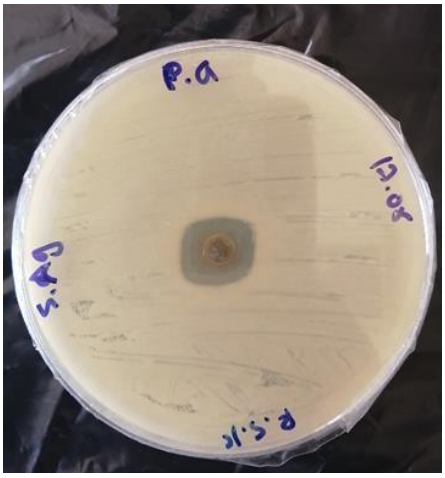

***Pseudomonas aeruginosa* (ATCC 19606)**

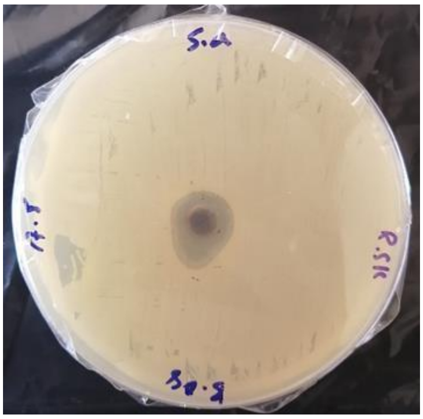

***Staphylococcus aureus* (ATCC 25923)**

Figure S3. Antimicrobial activity of silver carbonate nanoparticles prepared from the biomass of strain *Saccharopolyspora erythraea* S26.

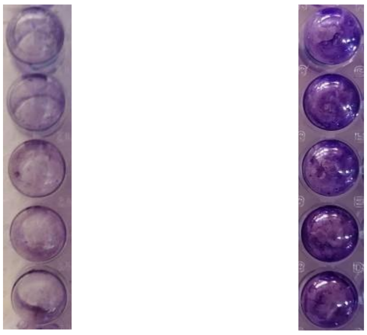

BioAg_2CO_3NPs Negative control

Figure S4. Microtiter plate biofilm assay for the evaluation of the antibiofilm activity of Bio-Ag_2CO_3NPs against the biofilm formed by strain *Klebsiella pneumoniae* kp6 strain

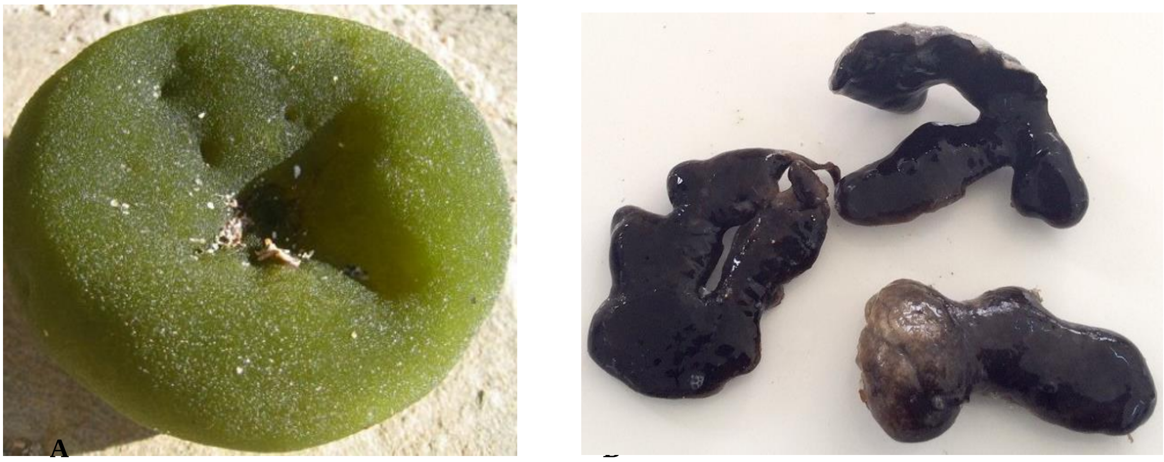

Figure S5. Marine samples used for Actinobacteria isolation. A : Marine algae Codium bursa; B : Marin sponge *Chondrosia reniformis*.

Table S1. Antimicrobial activity (mm) of *Actinobacteria* strains against different pathogenic microorganisms.

Origine of isolates	Isolates	*E. coli*	*K. pneumoniae*	*P. aeruginosa*	*S. aureus*	*M. luteus*	*B. cereus*	*L. monocytogenes*	*C. albicans*
Marine sediment	M51	-	8	-	-	-	21	-	15
	M52	-	-	-	-	16	-	-	8.5
	BS4	-	-	-	-	8.5	-	-	-
	BC8	-	-	-	8	-	-	-	-
	M2S9	-	-	8	-	-	-	-	-
	M2S10	-	-	-	-	-	17	-	14
	M2S12	-	-	-	12	-	-	-	-
	M19	-	-	-	11	-	-	-	-
	MHB2	-	-	-	9	10	-	-	9
	RHS22	-	-	-	-	-	-	-	10
	S26	-	-	-	31	21.5	29	-	20.5
	M2S27	-	-	-	-	-	8	-	-
	M2S28	-	-	-	-	-	12	-	12
	M29	-	-	-	11	-	9	-	10
	M2S30	-	-	-	12.5	-	-	-	11
	M5S34	-	-	-	-	9.5	-	-	8.5
	CS3	-	-	-	-	-	13	-	-
	CC54	-	-	-	-	15.5	-	-	-
	RR56	-	-	-	16	-	12	-	12.5
	RR57	-	-	-	8	-	15	-	19
	M2S59	-	-	-	-	-	9	-	11
	MRH94	-	-	-	30	29.5	28.5	-	21.5
	M2S108	-	-	-	-	-	-	-	10.5
	CS114	-	-	-	-	19	-	-	-
	M2S115	-	-	-	-	-	13	-	14
	MS44	-	-	-	20.5	-	12	-	19
	M2C6	-	-	-	-	-	16	-	-
	CS7	-	-	-	8.5	-	-	-	-
	CS16	-	-	-	-	-	10	-	-
	CS58	22	-	-	-	27	-	-	20
Marine algae	SW1V5	-	-	-	-	-	-	-	13.5
	W7V9	-	-	9	-	-	-	-	11.5
	W3V10	-	-	10	9	-	-	-	11
	W4V12	13.5	16.5	11	18.75	15.75	13	13.5	20
	V4	-	-	-	-	-	-	-	11.25
	S17	-	-	-	11.5	-	13	-	-
	W6V18	-	-	-	7.5	-	-	-	7
Marine sponge	SA6M7	-	-	10	-	-	-	-	-
	A6M14	-	-	-	8.75	-	-	-	7
	S23	-	-	-	14	-	9	-	13

Table S2. Molecular identification of the 18 selected active strains, based on 16S rRNA gene sequencing.

Isolates	Clusters	Closest reference species	Similarity (%)
S17	Cluster I	*Streptomyces spinoverrucosus*	99.72
MRH94		*Streptomyces luridiscabiei*	99.86
MS44		*Streptomyces hydrogenans*	99.46
M29		*Streptomyces artemisiae*	99.65
M2S28		*Streptomyces peucetius*	99.72
W3V10		*Streptomyces albogriseolus*	100
W4V12		*Streptomyces lavendofoliae*	99.88
W7V9		*Streptomyces pactum*	100
M51		*Streptomyces hydrogenans*	99.49
MHB2		*Streptomyces qinglanensis*	99.67
W6V18		*Streptomyces qinglanensis*	99.25
CS58	Cluster II	*Nocardiopsis terrae*	99.72
RR56		*Nocardiopsis terrae*	99.65
M52		*Nocardiopsis halotolerans*	100
RR57		*Nocardiopsis sinuspersici*	99.46
S23		*Nocardiopsis dassonvillei subsp. dassonvillei*	99.74
M2S10		*Nocardiopsis synnemataformans*	99.89
S26	Cluster III	*Saccharopolyspora erythraea*	99.87

Table S3. Characteristics of the S26 strain and the closest species within the genus *Saccharopolyspora*. 1. S26 ; 2. *Saccharopolyspora erythrea* DSM 40517^{T} (AM420293). 3. *Saccharopolyspora spinosporotrichia* DSM 44350^{T} (Y09571).

**Characters**	**1**	**2**	**3**
**Spores arrangement**	Open spirals	Open spirals	Spirals
**Spores ornamentation**	Spiny	Spiny	Spiny
**Substrate mycelium**	Branched	Branched	Fragmented
**Color**			
Substrate mycelium	Brown beige	Bright red	Beige
Aerial mycelium	Beige	White rose	White
Soluble pigment	Orange-brown		Pastel yellow
**Growth on sole carbon sources**			
Glucose	+	+	+
Arabinose	+	+	+
Saccharose	+	+	+
Xylose	+	+	+
Inositol	+	+	+
Mannose	+	+	+
Fructose	+	+	+
Rhamnose	+	+	+
Raffinose	-	+	+
Cellulose	-	+	+
**NaCl tolerance**	(0 - 5)%, Opt 0%	(0 - 5)%, Opt 0%	(0 - 7)%, Opt 0%
**Nitrate reductase**	-	+	-
**Ureas**	+	+	+

Table S4. Peak list of silver carbonate nanoparticles biosynthesis using the biomass strain S26.

Pos. [°2θ]	Height [cts]	FWHM Left [°2θ]	d-spacing [Å]	Rel. Int. [%]
17.6239	1446.11	0.1956	5.02833	18.26
19.5154	5645.86	0.2075	4.54503	71.31
30.3374	1502.61	0.8822	2.94387	18.98
30.6912	2351.64	0.1717	2.91073	29.70
33.8752	7917.86	0.7632	2.64408	100.00
35.5840	549.53	0.1120	2.52092	6.94
39.5070	1844.61	0.2524	2.27917	23.30
43.5341	577.84	0.8319	2.07721	7.30
48.5342	1515.54	0.7830	1.87425	19.14
53.0702	1022.72	0.2831	1.72424	12.92
54.3468	459.05	0.3933	1.68672	5.80
60.6894	1619.10	0.7809	1.52474	20.45
67.2534	237.20	0.5522	1.39099	3.00
71.5640	261.10	0.5183	1.31742	3.30
